# Epidemiology and short-term outcomes of acute kidney injury among patients in the intensive care unit in Laos: a nationwide multicenter, prospective, and observational study

**DOI:** 10.1186/s12916-020-01645-3

**Published:** 2020-07-14

**Authors:** Noot Sengthavisouk, Nuttha Lumlertgul, Chanmaly Keomany, Phonepadith Banouvong, Phetvilay Senavong, Sidavone Sayyaphet, Sakountala Binbundith, Win Kulvichit, Sadudee Peerapornratana, Kearkiat Praditpornsilpa, Kriang Tungsanga, Somchai Eiam-Ong, Nattachai Srisawat

**Affiliations:** 1 Department of Nephrology, Division of Critical Care, Mittaphab Hospital, Vientiane Capital, Laos; 2 Division of Nephrology, Department of Medicine, Faculty of Medicine, King Chulalongkorn Memorial Hospital, Chulalongkorn University, Bangkok, 10330 Thailand; 3 Excellence Center for Critical Care Nephrology, King Chulalongkorn Memorial Hospital, Bangkok, Thailand; 4 Critical Care Nephrology Research Unit, Chulalongkorn University, Bangkok, Thailand; 5 Division of Critical Care, Luangprabang Provincial Hospital, Luangprabang, Laos; 6 Division of Critical Care, Savannakhet Provincial Hospital, Savannakhet, Laos; 7 Division of Critical Care, Mahosot Hospital, Vientiane Capital, Laos; 8 Division of Critical Care, Champasak Provincial Hospital, Champasak, Laos; 9 Department of Laboratory Medicine, Faculty of Medicine, Chulalongkorn University, Bangkok, Thailand; 10 Academy of Science, Royal Society of Thailand, Bangkok, Thailand; 11 Tropical Medicine Cluster, Chulalongkorn University, Bangkok, Thailand; 12 Excellence Center for Critical Care Medicine, King Chulalongkorn Memorial Hospital, Bangkok, Thailand

**Keywords:** Acute kidney injury, Epidemiology, Intensive care unit, Laos

## Abstract

**Background:**

Acute kidney injury (AKI) has become a global health issue. Little is known about the disease burden in Laos. We aimed to evaluate the burden and outcomes of AKI as well as assess the availability of AKI treatment in Laos.

**Methods:**

We performed a multicentric prospective observational study in adult patients who had been admitted to 5 intensive care units (ICU) in Laos. The data was serially collected on the first 28 days of ICU admission. Patients were diagnosed by the KDIGO 2012 criteria for AKI. We used AKI occurrence as the primary outcome and explored risk factors on the development and outcomes of AKI.

**Results:**

We enrolled 1480 patients from 5 ICU centers across Laos from January to December 2016. After excluding patients with end-stage renal disease and those with incomplete data, AKI occurred in 508 of the 1460 enrolled patients (34.8%). Overall, the rates of maximum AKI staging were 4% for stage 1, 10.3% for stage 2, and 20.5% for stage 3. Risk factors for AKI were older age, obesity, cardiovascular diseases, respiratory diseases, renal diseases, oncologic diseases, and chronic kidney diseases. Only 1.8% of all participants received RRT. The mortality rate was 28.4% in non-AKI patients compared to 44.5% in AKI patients, which increased according to the stage of AKI (stage 1, 4.9%; stage 2, 28.3%; stage 3 66.8%; *P* < 0.001). There were 13.6% who were discharged against medical advice.

**Conclusions:**

AKI is a huge burden in Laos with under-recognition and poor outcomes.

## Background

Acute kidney injury (AKI) is a global burden resulting in increased morbidity and mortality [1]. In developed countries, AKI epidemiology has experienced improved prevention, diagnosis, and treatment [2]. However, AKI epidemiology in developing countries is poorly described. The International Society of Nephrology (ISN) conducted a “Global Snapshot” about AKI in 2014, where most cases (45%) were from low and lower middle-income countries [3]. Each year, AKI affects approximately 13.3 million individuals globally, with 85% of those affected living in developing countries and about 1.7 million resulting in death every year [4].

AKI in Asia is particularly challenging. The pooled incidence of AKI in the hospitalized population varied from 9 to 31% across regions [5, 6]. Causes include renal hypoperfusion, nephrotoxic drugs, urinary tract obstruction, envenomation and plant toxins, and obstetric complications; some of these are potentially preventable [7, 8]. Moreover, low availability of resources and inadequate health infrastructure are associated with poor recognition, underreporting, prevention, and treatment of AKI [9].

Laos is a country in the Southeast Asia region with a land area of 236,800 km^2^. It is ranked 139th on the Human Development Index (HDI), indicating medium development [10]. In 2011, it was classified as a low middle-income country (LMIC) by the World Bank GNI classification system, with a gross national income (GNI) of US $2408 per capita by 2016 [11]. Its population was 7.01 million in 2018 [12]. There are a total of 5 trained nephrologists in Laos, meaning approximately 1.45 nephrologists per million people, substantially less than the 2.28 physicians per 1000 people recommended by the WHO [13]. The absence of a unified electronic health management information system is a major barrier to consolidated national statistics for any disease, including AKI. Therefore, we designed a prospective multicenter study of critically ill adults treated in 5 hospitals in 2015 to illustrate the burden of AKI, as well as its characteristics and outcomes.

## Methods

**Fig. 1:**
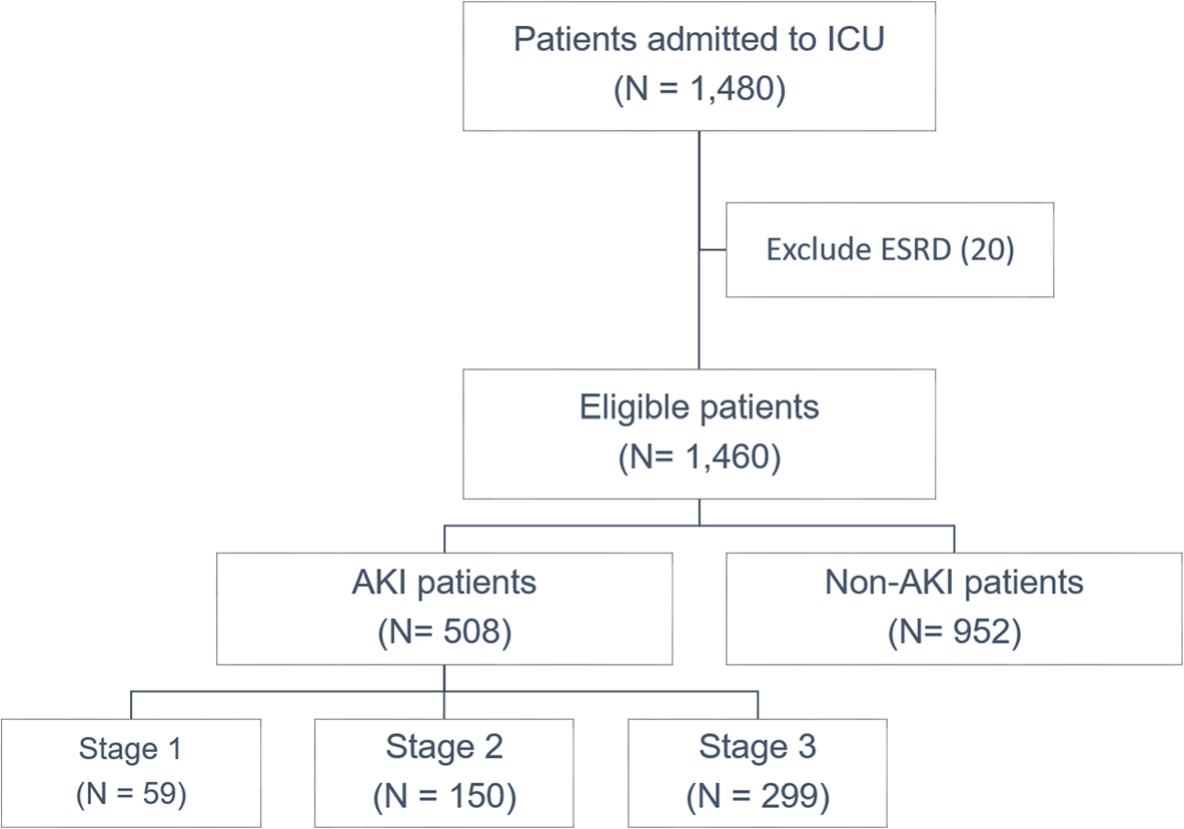
Study flowchart

We conducted a prospective multicenter observational study in 5 ICUs across various regions in Laos. Two hospitals were university hospitals under the Ministry of Health Regulation. Three hospitals were provincial hospitals under the jurisdiction of the local provincial government. All were tertiary referral hospitals, covering 3,188,100 people, which is approximately 44% of the overall Laos population [12]. All hospitals except Mahosot Hospital could provide dialysis. We enrolled patients who were > 18 years old and admitted to the participating ICUs from January 2016 to December 2016. Patients with end-stage renal diseases (ESRD) on chronic dialysis were excluded from our analysis (Fig. 1). For patients with multiple admissions, we collected data from only the first admission. The study protocol was reviewed by the National Ethics Committee for Health Research (no. 072 NIOPH/NECHR), and the need for informed consent was waived. This study was a part of the Southeast Asia-Acute Kidney Injury (SEA-AKI) study previously published by Srisawat et al. [6].

### Data collection

We collected data by registration in an electronic web-based format. Demographic, clinical, and laboratory data were recorded. Demographic data included age, gender, timing of hospital and ICU admission, comorbidities, and primary diagnosis at ICU admission. Clinical data included Acute Physiology and Chronic Health Evaluation (APACHE) II score at ICU admission, Sequential Organ Failure Assessment (SOFA) scores for the first 3 days, fluid balance status, use of a mechanical ventilator, vasopressor use, and renal replacement therapy (RRT). Laboratory data included blood urea nitrogen level and serum creatinine level if available. The data was collected sequentially every day for the first 7 days, after which it was collected weekly on days 14, 21, and 28. The primary outcome was AKI (any stage).

### Definitions

The diagnosis of AKI was determined by the KDIGO criteria [14]. For the baseline serum creatinine, we used the most recent available serum creatinine before hospital admission within 1 year. If the patients had no available data for baseline serum creatinine, then we estimated baseline serum creatinine using the lowest value between the serum creatinine at the time of hospital admission (admission serum creatinine) and the back calculation of serum creatinine from the Modification of Diet in Renal Disease (MDRD) equation using a glomerular filtration rate (GFR) of 75 mL/min/1.73m^2^ (MDRD serum creatinine) [15] as recommended in the KDIGO guideline [14]. For the urine output criteria, we modified the criteria to be cumulative 24-h urine output. We defined patients who had urine output > 0.5 mL/kg/h as being “no AKI,” 0.3–0.5 mL/kg/h as KDIGO stage 2, and < 0.3 mL/kg/h as KDIGO stage 3 [15]. We used the body weight value before hospital admission to calculate the rate of urine flow. If no known body weight was available, we used ideal body weight which was calculated from height (cm)-100 in males or height (cm)-110 in females.

Both the primary diagnosis for ICU admission and the etiologies of AKI were determined by the study team. We used the International Classification of Diseases, 10th Revision coding to classify the primary diagnosis of ICU admission. The definition of renal-related disease includes AKI, glomerular diseases, electrolyte imbalances related to renal disorders, and stones in the urinary tract system. However, we excluded patients who had AKI as a primary diagnosis in our AKI risk factor analysis.

All participants in this study were Asian. Body mass index (BMI) class was defined using the World Health Organization Expert Consultation criteria [16]. Community-acquired AKI (CA-AKI) was defined as AKI onset on hospital admission and within 48 h after admission. Hospital-acquired AKI (HA-AKI) was defined as AKI onset 48 h after admission. We have excluded the cases who were admitted to general ward first. Discharge against medical advice (AMA) is defined when a patient chose to leave the hospital before the treating physician recommends discharge.

### Statistical analysis

Categorical data was presented as counts and percentages. Continuous data was presented as mean and standard deviation (SD) if normally distributed or median with interquartile range if non-normally distributed. The Kaplan-Meier survival curves were also generated for each AKI class. The chi-square test of independence was used to compare the proportions of different types of patients between patients by AKI status, whereas analysis of variance (ANOVA) was used to compare continuous patient characteristics by AKI status. As this was a multicenter study with centers representing clusters, binary logistics mixed effect regression was used to identify the risk factors for AKI [17]. This enabled us to adjust for the hospital (cluster)-specific random effect. We also investigated both the random intercept and random coefficients model. We chose the random intercept model as it had a lower Akaike information criterion and therefore a lower propensity to overfit to the sample. We ran multicollinearity diagnostics as part of our modeling process, with variance inflation factors above 5 indicating a concern. Factors showing an association with AKI at a level of *P* < 0.20 were included in the multivariable analysis. All analyses were conducted using Stata 14.0. A *P* < 0.05 was considered to be statistically significant.

## Results

### ICU characteristics

In total, there were 50 ICU beds among participating ICUs, each site having from 7 to 14 beds with the average of 10 beds per ICU. The enrollment period for each center was approximately 1 year. Every center started collecting data at the same time. The approximate admission rate across centers was 25 patients per month. All patients were admitted to mixed ICUs.

### Patient characteristics

After excluding ESRD patients or those with missing values, 1460 patients were included in the final analysis (Fig. 1). The average age of patients was 53.4 years, with 42.12% being female. In total, 1065 (72.95%) patients were in the optimal BMI class, 280 (19.18%) were either overweight or obese, and 115 (7.88%) patients were classified as underweight. The most common diagnosis for ICU admission was surgical-related disease (26.10%). Mean APACHE II score was 16.6 ± 8.6. The majority of the patients’ medical expenses comprised out-of-pocket payments (84.1%). Baseline creatinine was available in 193 patients (13.2%). Three hundred and sixty-five patients (25%) had serum creatinine measured two or more times during admission. We have 260 ICU cases who were admitted to general wards before ICU admission and 1200 ICU cases who were admitted directly to the ICU. Baseline characteristics for all patients are shown in Table 1.

**Table 1:** Patient characteristics stratified by AKI status

	Non-AKI (*N* = 952)	AKI (***n*** = 508)	All (*N* = 1460)
Age (years)	50.9 (20.3)	58 (18)	53.4 (19.8)
Female	405 (42.5%)	210 (41.3%)	615 (42.1%)
Reimbursement			
Government officer	56 (5.9%)	31 (6.1%)	85 (5.8%)
Out-of-pockets	810 (85.2%)	417 (82.1%)	1227 (84.1%)
SS, private insurances	85 (8.9%)	60 (11.8%)	145 (9.9%)
BMI			
Underweight	79 (8.3%)	36 (7.1%)	115 (7.9%)
Normal	707 (74.3%)	358 (70.5%)	1065 (73.0%)
Overweight/obese	166 (17.4%)	114 (22.4%)	280 (19.1%)
Primary diagnosis			
Cardiovascular diseases	60 (6.3%)	48 (9.5%)	108 (7.4%)
Renal diseases	16 (1.7%)	49 (9.7%)	65 (4.5%)
Infectious diseases	157 (16.5%)	77 (15.2%)	234 (16.0%)
Gastrointestinal diseases	113 (11.9%)	58 (11.4%)	171 (11.7%)
Hematologic disease	13 (1.4%)	9 (1.8%)	22 (1.5%)
Respiratory diseases	101 (10.6%)	81 (15.9%)	182 (12.5%)
Neurologic diseases	150 (15.8%)	51 (10.0%)	201 (13.8%)
Endocrine diseases	34 (3.6%)	42 (8.3%)	76 (5.2%)
Rheumatologic diseases	2 (0.2%)	1 (0.2%)	3 (0.2%)
Oncologic diseases	8 (0.8%)	9 (1.8%)	17 (1.2%)
Surgical-related diseases	298 (31.3%)	83 (16.3%)	381 (26.1%)
Comorbidity			
HT	260 (27.3%)	179 (35.2%)	439 (30.1%)
DM	168 (17.6%)	160 (31.5%)	328 (22.5%)
CKD	25 (2.6%)	121 (23.8%)	146 (10%)
Cerebrovascular	23 (2.4%)	18 (3.5%)	41 (2.8%)
Malignancy	21 (2.2%)	15 (3%)	36 (2.5%)
CAD	11 (1.2%)	14 (2.8%)	25 (1.7%)
APACHE II score	15.6 (8.5)	18.3 (8.5%)	16.6 (8.6%)
Non-renal SOFA score	5.4 (3.2)	5.5 (3.5)	5.4 (3.3)
Vasopressors	201 (21.1%)	153 (30.1%)	354 (24.3%)
Mechanical ventilation	260 (27.3%)	98 (19.3%)	358 (24.5%)
Urine output (mL/day)	1293.7 (872.1)	747.2 (731.5)	1103.6 (865.7)
Anemia^a^	315 (33.1%)	273 (53.7%)	588 (40.3%)
Percent of fluid accumulation^b^	0.42 (1.5)	0.79 (1.5)	0.55 (1.6)
Diuretics	99 (10.4%)	110 (21.7%)	209 (14.3%)

Data are mean (SD), *n* (%), or median (Q1, Q3), unless stated otherwise

*SS* social security, *BMI* body mass index, *HT* hypertension, *DM* diabetes mellitus, *CKD* chronic kidney disease, *CAD* coronary artery disease, *CVD* cerebrovascular disease, *APACHE II* Acute Physiology and Chronic Health Evaluation II, *SOFA* Sequential Organ Failure Assessment

All these parameters came from the first day of ICU admission

^1^*P* value from Fisher’s exact test instead of Chi-square test

^a^Anemia is defined as hemoglobin < 13.5 g/dL in men and < 12 g/dL in women

^b^Percent of fluid accumulation equals day 1 intake (L) − output (L)/baseline body weight (kg) × 100

### AKI rates and characteristics

Of the 1460 patients who were screened, 508 ICU patients (34.8%) were diagnosed with AKI during admission based on the KDIGO criteria (Fig. 1, Additional file: Table S1). AKI criteria were met in 197 (36.7%), 178 (35.0%), and 133 (26.3%) patients based on serum creatinine criteria alone, urine output criteria, and both criteria, respectively. Maximal AKI stage 3 (20.5%) was the most common, followed by stage 2 (10.3%) and stage 1 (4%). (Fig. 1, Additional file: Figure S1). Eighty-three percent of AKI patients were categorized as CA-AKI. Renal hypoperfusion was the leading cause of AKI. It was found 1.97%, 1.18%, and 0.2% of all patients’ AKI etiology were obstetrics-related causes, tropical diseases, and toxin/poisoning (Table 2).

**Table 2:** AKI etiology

	Non-severe (*n* = 209)	Severe (*n* = 299)	All AKI (*n* = 508)	*P* value
Sepsis	48 (23.0%)	61 (20.4%)	107 (27.5%)	0.49
Renal hypoperfusion^a^	114 (54.6%)	127 (42.5%)	241 (47.4%)	0.01
Toxin and poisoning^b^	0	1 (0.3%)	1 (0.2%)	0.40^1^
Trauma and surgery	21 (10.1%)	39 (13.0%)	60 (11.8%)	0.30
Systemic diseases^c^	1 (0.5%)	0	1 (0.2%)	0.41^1^
Genitourinary diseases^d^	4 (1.9%)	4 (1.3%)	8 (1.6%)	0.72^1^
Tropical infection^e^	4 (1.9%)	2 (0.7%)	6 (1.2%)	0.24^1^
Obstetric complications^f^	5 (2.4%)	5 (1.7%)	10 (2.0%)	0.75^1^
Other^g^	12 (5.7%)	60 (20.1%)	72 (14.2%)	< 0.001

^1^*P* value from Fisher’s exact test instead of chi-square test

^a^Renal hypoperfusion included hypotension, hemorrhage, volume depletion, and cardiorenal syndrome

^b^Toxin and poisoning included envenomation, snake bites, wasp or bee stings, and mushroom and pesticide ingestion

^c^Systemic diseases included connective tissue diseases such as systemic lupus erythematosus and vasculitis

^d^Genitourinary diseases included obstructive uropathy, renal stones, benign prostatic hyperplasia, and malignancy in the kidney, bladder, and ureteral system

^e^Tropical infectious disease included leptospirosis, malaria, scrub typhus, and dengue fever

^f^Obstetric complications included eclampsia or severe preeclampsia, septic abortion, postpartum hemorrhage, ruptured placenta, and hyperemesis gravidarum

^g^Others included patients with missing AKI etiologies

Multivariable binary logistics mixed effect modeling (only included the data on day 1 of ICU admission and excluded patients who had AKI as the primary diagnosis for ICU admission) revealed older age and overweight/obesity associated with AKI. Primary diagnoses of cardiovascular diseases, renal diseases, respiratory diseases, oncologic diseases, and underlying CKD were risk factors for AKI development. Anemia, percentage of fluid accumulation, and diuretic use were also associated with developing AKI (Table 3).

**Table 3:** Risk factors for AKI development using mixed logistic regression clustering by sites

	Adjusted OR (95% CI)	*P* value
Age, 10-year increment	1.14 (1.06, 1.24)	0.001
Reimbursement		
Government officer	0.66 (0.35, 1.26)	0.21
Out-of-pockets	0.76 (0.51, 1.15)	0.19
SS, private insurances	Reference	
BMI		
Underweight	1.01 (0.64, 1.61)	0.96
Normal	Reference	
Overweight/obese	1.59 (1.15, 2.19)	0.005
Primary diagnosis		
Cardiovascular diseases	1.79 (1.03, 3.11)	0.04
Renal diseases	3.60 (1.74, 7.45)	0.001
Infectious diseases	1.45 (0.96, 2.21)	0.08
Gastrointestinal diseases	1.30 (0.81, 2.10)	0.28
Hematologic disease	2.36 (0.88, 6.34)	0.09
Respiratory diseases	1.68 (1.03, 2.74)	0.04
Neurologic diseases	1.04 (0.64, 1.69)	0.87
Endocrine diseases	1.68 (0.86, 3.28)	0.13
Rheumatologic diseases	0.84 (0.05, 15.82)	0.91
Oncologic diseases	4.81 (1.67, 13.87)	0.004
Surgical-related diseases	Reference	
Comorbidity		
HT	0.99 (0.70, 1.40)	0.96
DM	1.28 (0.92, 1.78)	0.14
CKD	6.57 (3.98, 10.83)	< 0.001
CAD	1.39 (0.54, 3.61)	0.50
CVD		
Malignancy		
APACHE II score	1.00 (0.98, 1.03)	0.89
Non-renal SOFA score	1.03 (0.97, 1.10)	0.56
Mechanical ventilation	0.90 (0.64, 1.28)	0.56
Anemia^a^	1.39 (1.03, 1.87)	0.03
Fluid accumulation (per 1% increase)	1.28 (1.17, 1.39)	< 0.001
Diuretics	1.60 (1.08, 2.37)	0.02

All these parameters came from the first day of ICU admission

Exclude 14 patients who had AKI as the primary diagnosis for ICU admission

*SS* social security, *HT* hypertension, *BMI* body mass index, *DM* diabetes mellitus, *CKD* chronic kidney disease, *CAD* coronary artery disease, *CVD* cerebrovascular disease, *APACHE II* Acute Physiology and Chronic Health Evaluation II, *SOFA* Sequential Organ Failure Assessment

^a^Anemia is defined as hemoglobin < 13.5 g/dL in men and < 12 g/dL in women

Focusing on severe AKI (AKI stage 3), primary diagnoses of renal-related diseases and neurologic diseases were more common in severe AKI than mild to moderate AKI. Multivariate analyses revealed only renal diseases, mechanical ventilation, and CA-AKI were associated with severe AKI (Additional file: Table S2).

We had 26 patients (1.8% of 1460 ICU patients, 5.1% of 508 AKI patients) who received RRT (Additional file: Table S3). The proportions of RRT in each center ranged from 0 to 21.1%. Intermittent hemodialysis was the only modality of RRT in our study. Patients who received RRT were older and had a higher percentage of fluid accumulation, higher diuretic use, and higher proportions of anemia, but had lower non-renal SOFA scores. None of the patients with mechanical ventilation was found in RRT patients (Additional file: Table S4, Table S5). Indications for RRT were azotemia (100%), hyperkalemia (46.15%), oliguria (42.31%), and acidosis (11.54%).

### AKI outcomes

The overall mortality in this cohort was 35.1%. The mortality rate was 44.49% in AKI compared with 28.36% in non-AKI patients (risk ratio 1.56, 95% 1.36–1.80, *P* < 0.001). The hospital mortality rate increased according to the stage of AKI (Additional file: Table S6). There were 13.6% of patients who were discharged AMA. Patients who died or were discharged AMA were more likely to pay out-of-pocket expenses, had underlying diseases of hypertension and diabetes, and had higher severity scores, mechanical ventilator use, and a higher percentage of fluid accumulation. However, they had lower vasopressor use, lower urine output, and lower diuretic use in addition to being less likely to receive RRT (Table 4).

**Table 4:** Patient characteristics stratified by discharge status among AKI patients (*n* = 508)

	Survive (*n* = 207)	Die or AMA (*n* = 301)	*P* value
Reimbursement			0.02
Government officer	16 (7.7%)	15 (5.0%)	
Out-of-pockets	158 (76.3%)	259 (86.1%)	
SS, private insurances	33 (15.9%)	27 (9.0%)	
Primary diagnosis			0.003
Infectious diseases	22 (10.6%)	55 (18.3%)	0.02
Gastrointestinal diseases	34 (16.4%)	24 (8.0%)	0.003
Neurologic diseases	14 (6.8%)	37 (12.3%)	0.04
Comorbidity			
HT	60 (29.0%)	119 (39.5%)	0.01
DM	46 (22.2%)	114 (37.9%)	< 0.001
AKI staging			0.03
1	33 (15.9%)	26 (8.6%)	
2	63 (30.4%)	87 (28.9%)	
3	111 (53.6%)	188 (62.5%)	
AKI etiology			
Renal hypoperfusion	113 (54.6%)	128 (42.5%)	0.007
APACHE II score	15.4 (6.9)	20.3 (8.9)	< 0.001
Non-renal SOFA score	3.4 (2.2)	7.0 (3.4)	< 0.001
Vasopressors	172 (83.1%)	219 (72.8%)	0.007
Mechanical ventilation	14 (6.8%)	84 (27.9%)	< 0.001
RRT	18 (8.7%)	8 (2.7%)	0.002
Urine output (mL)	600 (400, 1000)	400 (200, 1000)	< 0.001
Diuretics	72 (34.8%)	38 (12.6%)	< 0.001

Data are mean (SD), *n* (%), or median (Q1, Q3), unless stated otherwise

Showed only the significant parameters

*AMA* against medical advice, *SS* social security, *HT* hypertension, *BMI* body mass index, *DM* diabetes mellitus, *CKD* chronic kidney disease, *CAD* coronary artery disease, *CVD* cerebrovascular disease, *CA-AKI* community-acquired AKI, *HA-AKI* hospital-acquired AKI, *APACHE II* Acute Physiology and Chronic Health Evaluation II, *SOFA* Sequential Organ Failure Assessment, *RRT* renal replacement therapy

**Fig. 2:**
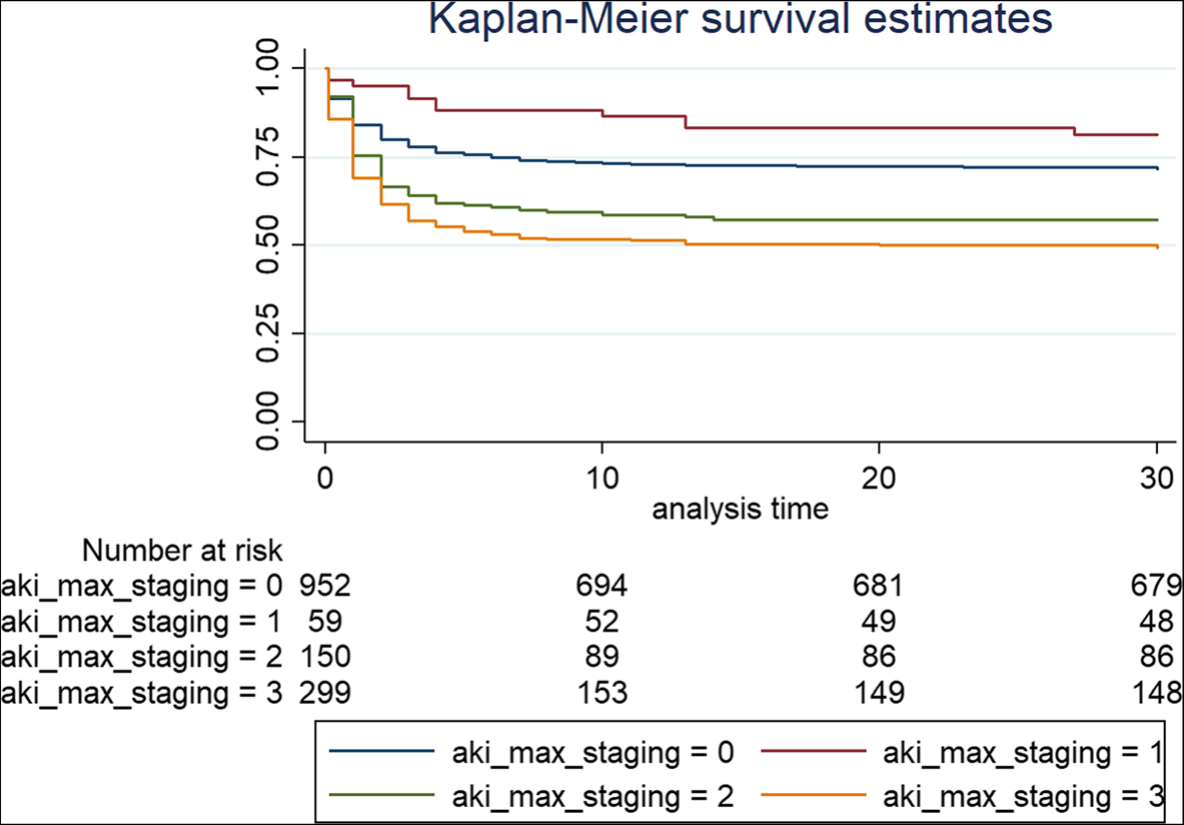
The Kaplan-Meier survival curves for each acute kidney injury stage on 30-day hospital mortality

In terms of comparing the survival of patients with different stages of AKI, Fig. 2 provides the Kaplan-Meier curves for each class for 30-day in-hospital mortality. First, we grouped discharges AMA with deceased patients. The hazard ratio (HR) in AKI stage 1 was comparable with non-AKI patients (HR 0.60, 95% CI 0.33–1.10, *P* = 0.098), but substantially higher risk was apparent in AKI stages 2 (HR 1.61, 95% CI 1.22–2.11, *P* = 0.001) and 3 (HR 2.03, 95% CI 1.67–2.48). Among AKI patients, higher AKI staging had poorer survival than lower AKI staging (log rank *P* < 0.001). The Kaplan-Meier curves for hospital mortality after exclusion of discharges AMA are shown in Additional file: Figure S2. Risk factors for death or AMA are shown in Additional file: Table S7. Twenty-one AKI cases were alive or HAMA at ICU discharge with requiring dialysis.

Focusing on discharges AMA among AKI patients, seventy-five patients who were discharged AMA were older, had milder AKI staging, and had AKI from sepsis compared with survivors and non-survivors. Mean APACHE II score was 16.2 ± 7.3, which was higher than survivors, but lower than non-survivors. None of those patients received RRT, and the median hospital length of stay was 3 (IQR 2, 6) days compared with 7 (IQR 5, 11) days for survivors and 2 (IQR 1, 5) days in non-survivors (Table S6).

## Discussion

This is the first report of AKI incidence, causes, and outcomes in Laos. AKI occurred in 34.8% of critically ill patients. The most common etiologies were renal hypoperfusion, sepsis, and trauma. Among AKI patients, only 5.1% received RRT. The only available mode of RRT is intermittent dialysis. At hospital discharge, 52.4% survived, 33.9% died, and 13.6% were discharged AMA.

The incidence of AKI in Laos is lower than other studies in Southeast Asia [6, 18]. Notably, only 25% of patients in our cohort had repeat Cr measurements during hospital admission, which was identical to China’s numbers nationwide, but much lower than those from developed countries (63.2–67.6%) [19–21]. The prevalence of AKI in our study could have substantially underestimated the actual national burden of AKI. We have missing data of serum creatinine on day 1 of ICU admission for 18%, day 2 for 71%, day 3 for 82%, day 4 for 84%, day 5 for 84%, day 6 for 85%, and day 7 for 80%. Decreased percentage of serum creatinine on subsequent days might be partially explained by the limited resource to check daily serum creatinine and by short ICU length of stay (median 3, IQR 2, 4) days. Lack of baseline Cr in most patients could also pose a big challenge for the evaluation of AKI prevalence and staging.

Renal hypoperfusion is the most common etiology here, which is the leading cause of AKI in low-income countries [22, 23]. The relatively low percentage of fluid accumulation, i.e., 0.79% versus 0.42%, in AKI and non-AKI patients, might reflect under-resuscitation as an important risk factor of AKI. Our study only found 0.27% of tropical infection. This is probably from under-recognition from the lack of specific and point-of-care tests, which might lead to underestimation of the true burden of tropical diseases.

Most patients had maximum AKI stage 2 or 3, contrary to AKI from high-income countries, where AKI stage 1 is more common [1]. High severity of AKI on admission might reflect delayed recognition and admission, while in-hospital monitoring allows practitioners to detect HA-AKI earlier. Only 5.1% of all AKI patients and only 8% of AKI stage 3 patients received RRT. Patients who received RRT were younger, did not use any mechanical ventilator, and had lower non-renal SOFA score, which could result in selection bias. Recent report from the WHO showed that population coverage by any prepayment health financing scheme was limited to around 33% of the entire population in Laos [24]. In our study, out-of-pocket payments were as high as 84% for all patients. Limited staff and RRT equipment, and financial issues might factor into the selection of relatively healthier patients for RRT. Alarmingly, the 28-day mortality rate was 28.36% in non-AKI patients versus 44.49% in AKI patients, higher than the approximately 10–20% in western countries [25, 26]. The high mortality across non-AKI and each AKI stage might represent inadequate overall care for critically ill patients.

Our study showed a very high amount of total discharges AMA. Previous studies in developed countries reported 1–2% discharges AMA [27], but no studies have explored AMAs in resource-limited settings. Patients who chose AMA tended to be older, were not covered by health reimbursement schemes, and had milder AKI stage as well as moderate APACHE II score with hospital stays of short duration. The high percentage of AMAs may be explained by patients forgoing health services due to financial burdens, as demonstrated by low healthcare utilization rates, which may result in increased levels of preventable mortality and disabilities. The other reasons might be from a lack of health literacy, or religious and cultural beliefs. Unfortunately, we were unable to trace the outcomes after discharge due to the lack of a national registry or the reasoning behind decisions made against medical advice.

There were several limitations in our study including lack of status after discharge, lack of renal recovery data, and lack of long-term outcomes. Our data was collected from 5 tertiary care ICUs distributed across various regions in Laos. It did not include local hospitals and could have missed patients who did not reach any hospitals for treatment. In our study, only 13% of the participants had true baseline serum creatinine. We recognized the possibility of AKI overestimation from the use of MDRD back calculation in the CKD group. Our database includes CKD status before admission. If a patient had a history of CKD, we used the first available serum creatinine as the baseline serum creatinine, not the MDRD back calculation. Environmental risk factors such as contaminated water, inadequate sanitation, or use of traditional medicines could not be delineated. Finally, the risk of residual confounding factors and statistically significant findings from a large sample size cannot be ruled out. Despite these limitations, our study is the first prospective data aspiring to obtain the current AKI situation in Laos systematically to be able to raise awareness and drive the health policy to improve AKI care [24]. For further studies, the lack of RRT should be explored for reasons such as cultural beliefs, socioeconomic status, or futility. Discharge AMA should be further clarified for causes.

## Conclusion

In conclusion, this study fills the gap concerning AKI epidemiology in Laos, a LMIC in Southeast Asia, which has never previously been documented. The lack of adequate laboratory measurement is demonstrated here as a limitation to AKI diagnosis. The most common cause of AKI is associated with renal hypoperfusion. Thus, inexpensive interventions such as fluid therapy can be implemented by simple protocol-based care in local settings. RRT is severely inadequate in terms of both numbers and modalities. Now that AKI burden is identified, strategies to improve national health policy should be encouraged for sustainable improvement in AKI education and care to achieve the 0by25 goal.

## Supplementary information

**Additional file 1: Table S1.** Cumulative prevalence of AKI by sites (*N*=1,460). **Table S2.** Patients characteristics stratified by severity of AKI (*N*=508) using mixed logistic regression clustering by sites. **Table S3.** Proportion of RRT in AKI patients (*N*=508). **Table S4** Patients characteristics stratified by RRT in severe AKI (*N*=299). **Table S5.** Univariate and multivariate analysis of potential risk factors for renal replacement therapy (RRT) in patients with stage 3 (*n*= 299) using mixed logistic regression clustering by sites. **Table S6.** Patients characteristics characterized by hospital discharge status in all AKI patients (*n* = 508). **Table S7.** Univariate and multivariate analysis of potential risk factors for death or HAMA in AKI patients (*n* = 508) using mixed logistic regression clustering by sites. **Figure S1.** Distribution of patients by maximum AKI stage for each hospital type. Y-axis represents the percentage of patients. X-axis represents hospital type. The number on top of the bars is the raw count. **Figure S2.** Kaplan-Meier survival curves for each AKI stage on hospital mortality after excluding hospital discharge against medical advice (*n* = 1,261; Log rank *p* < 0.001)**. Figure S3.** Kaplan-Meier survival curves for each AKI stage on ICU mortality (*n* = 1,460; Log rank p < 0.001)**. Figure S4.** Kaplan-Meier survival curves for each AKI stage on hospital mortality after excluding hospital discharge against medical advice (*n* = 1,261; Log rank *p* < 0.001).

## Data Availability

Data and materials are available upon reasonable request to the corresponding author.
