# Supplementary material for: Epidemiology and short-term outcomes of acute kidney injury among patients in the intensive care unit in Laos: a nationwide multicenter, prospective, and observational study
Source: BMC Med. 2020 Jul 14;18:180. doi: 10.1186/s12916-020-01645-3 (PMC7358323; doi:10.1186/s12916-020-01645-3)

**Supplementary material**

**Table S1** Cumulative prevalence of AKI by sites (N=1,460)

| **Site** | **Population** | **ICU beds** | **Number of included patients** | **Number of AKI patients** | **Prevalence of AKI**  **(95% CI)** |
| --- | --- | --- | --- | --- | --- |
| Mahosot hospital | 927,700 (Vientiane) | 7 | 217 | 72 | 33.2  (23.9-33.9) |
| Mitraphap hospital | 927,700  (Vientiane) | 12 | 510 | 175 | 34.3  (27.2-39.7) |
| Savannakhet hospital | 1,053,900 | 14 | 288 | 111 | 38.5  (30.3-38.6) |
| Champasak hospital | 743,200 | 8 | 131 | 60 | 45.8  (33.1-44.3) |
| Luang Prabang hospital | 463,300 | 9 | 314 | 90 | 28.7  (37.4-54.4) |
| Overall | 3,188,100 | 50 | 1,460 | 508 | 34.8  (32.4-37.3) |

Ref: Laos statistics bureau <https://www.lsb.gov.la/en/#.XYClUCgzbD4>

**Table S2** Patients characteristics stratified by severity of AKI (N=508) using mixed logistic regression clustering by sites

| **Characteristics** | **Severity, n (%)** | | **Univariate** | | **Multivariate** |  |
| --- | --- | --- | --- | --- | --- | --- |
|  | **Non-severe (N=209)** | **Severe (N=299)** | **Unadjusted OR (95% CI)** | **P-value** | **Adjusted OR (95% CI)** | **P-value** |
| Age, 10-year increment^1^ | 57.0 (18.8) | 58.7 (17.4) | 1.03 (0.93, 1.14) | 0.57 |  |  |
| Female | 78 (37.1%) | 132 (44.2%) | 1.40 (0.96, 2.03) | 0.08 | 1.21 (0.80,1.83) | 0.36 |
| Reimbursement |  |  |  |  |  |  |
| - Government officer | 14 (6.7%) | 17 (5.7%) | 1.79 (0.72, 4.46) | 0.21 |  |  |
| - Out-of-pockets | 166 (79.4%) | 251 (84.0%) | 1.22 (0.68, 2.17) | 0.50 |  |  |
| - Social security, Private insurances | 29 (13.9%) | 31 (10.4%) | Reference |  |  |  |
| BMI |  |  |  |  |  |  |
| - Underweight | 15 (7.2%) | 21 (7.0%) | 1.09 (0.53, 2.25) | 0.81 |  |  |
| - Normal | 141 (67.5%) | 217 (72.6%) | Reference |  |  |  |
| - Overweight / obese | 53 (25.4%) | 61 (20.4%) | 0.98 (0.62, 1.56) | 0.94 |  |  |
| Primary diagnosis |  |  |  |  |  |  |
| - Cardiovascular diseases | 20 (9.6%) | 28 (9.4%) | 0.76 (0.40,1.44) | 0.40 |  |  |
| - Renal diseases | 8 (3.8%) | 41 (13.7%) | 4.04 (1.81,9.02) | 0.001 | 2.81 (1.16,6.80) | 0.02 |
| - Infectious diseases | 28 (13.4%) | 49 (16.4%) | 1.37 (0.81,2.30) | 0.24 |  |  |
| - Gastrointestinal diseases | 28 (13.4%) | 30 (10.0%) | 0.80 (0.45,1.41) | 0.44 |  |  |
| - Hematologic disease | 6 (2.9%) | 3 (1.0%) | 0.43 (0.10,1.82) | 0.25 |  |  |
| - Respiratory diseases | 32 (15.3%) | 49 (16.4%) | 1.19 (0.71,1.99) | 0.51 |  |  |
| - Neurologic diseases | 27 (12.9%) | 24 (8.0%) | 0.52 (0.28,0.97) | 0.04 | 0.56 (0.27,1.15) | 0.12 |
| - Endocrine diseases | 16 (7.7%) | 26 (8.7%) | 1.00 (0.51,1.98) | 1.00 |  |  |
| - Rheumatologic diseases | 0 | 1 (0.3%) | NA |  |  |  |
| - Oncologic diseases | 6 (2.9%) | 3 (1.0%) | 0.34 (0.08,1.42) | 0.14 | 0.53  (0.12, 2.40) | 0.41 |
| - Surgical related diseases | 28 (18.2%) | 45 (15.1%) | 0.82(0.50,1.36) | 0.45 |  |  |
| Comorbidity |  |  |  |  |  |  |
| - Hypertension | 66 (31.6%) | 113 (37.8%) | 1.26 (0.85, 1.88) | 0.25 |  |  |
| - DM | 52 (24.9%) | 108 (36.1%) | 1.61 (1.07, 2.42) | 0.02 | 1.44 (0.91,2.27) | 0.12 |
| - CKD | 34 (16.3%) | 87 (29.1%) | 2.05 (1.29, 3.25) | 0.002 | 1.00  (0.57,1.74) | 0.99 |
| - CAD | 5 (2.4%) | 9 (3.0%) | 1.00 (0.32, 3.16) | 0.99 |  |  |
| - CVD | 9 (4.3%) | 9 (3.0%) | 0.74 (0.28, 1.98) | 0.55 |  |  |
| - Malignancy | 10 (4.8%) | 5 (1.7%) | 0.39 (0.13, 1.20) | 0.10 |  |  |
| APACHE-II score | 16.4 (8.5) | 19.7 (8.1) | 1.05 (1.02, 1.07) | 0.001 | 1.02 (0.99,1.05) | 0.22 |
| Non-renal SOFA score | 5.1 (3.3) | 5.9 (3.6) | 1.04 (0.97, 1.12) | 0.23 |  |  |
| Vasopressors | 51 (24.4%) | 76 (25.4%) | 0.95 (0.61, 1.47) | 0.81 |  |  |
| Mechanical ventilation | 30 (14.4%) | 68 (22.7%) | 1.53 (0.93, 2.53) | 0.096 | 2.43 (1.33,4.45) | 0.004 |
| Anemia | 98 (46.9%) | 175 (58.5%) | 1.66 (1.65,2.41) | 0.007 | 1.22 (0.78,1.90) | 0.38 |
| AKI Etiology |  |  |  |  |  |  |
| - Sepsis | 48 (23.0%) | 61 (20.4%) | 0.88 (0.56,1.37) | 0.57 |  |  |
| - Renal hypoperfusion | 114 (54.6%) | 127 (42.5%) | 0.69 (0.47,0.99) | 0.046 | 0.83 (0.53, 1.29) | 0.41 |
| - Toxin and poisoning | 0 | 1 (0.3%) |  |  |  |  |
| - Trauma | 21 (10.1%) | 39 (13.0%) | 1.09 (0.61,1.96) | 0.77 |  |  |
| - Systemic disease | 1 (0.5%) | 0 |  |  |  |  |
| - Genitourinary | 4 (1.9%) | 4 (1.3%) | 0.89 (0.22,3.74) | 0.88 |  |  |
| - Tropical infection | 4 (1.9%) | 2 (0.7%) | 0.48 (0.09,2.72) | 0.41 |  |  |
| - Obstetric complications | 5 (2.4%) | 5 (1.7%) | 0.96 (0.26,3.55) | 0.95 |  |  |
| - Others | 12 (5.7%) | 60 (20.1%) | 3.35  (1.72,6.52) | <0.001 |  |  |
| AKI type* | (N=178) | (N=245) |  |  |  |  |
| - CA-AKI | 74 (41.6%) | 186 (75.9%) | 3.87 (2.50, 5.99) | <0.001 | 2.90 (1.85, 4.54) | <0.001 |
| - HA-AKI | 104 (58.4%) | 59 (24.1%) | Reference |  | Reference |  |
| Fluid accumulation | 0.66  (-0.17,1.33) | 0.82  (0.17,1.62) | 1.13 (1.00,1.28) | 0.048 | 1.06  (0.93, 1.22) | 0.38 |
| Diuretics | 35 (16.8%) | 75 (25.1%) | 2.48 (1.50,4.09) | <0.001 | 1.68  (0.97, 2.91) | 0.06 |

Data are mean (SD), n (%), or median (Q1,Q3), unless stated otherwise.

All these parameters came from the first day of ICU admission

BMI: body mass index, DM: diabetes mellitus, CKD: chronic kidney disease, CAD: coronary artery disease, CVD: cerebrovascular disease, APACHE II: acute physiologic and chronic health evaluation II, SOFA: sequential organ failure assessment, CA-AKI: community-acquired AKI, HA-AKI: hospital-acquired AKI

* Excluded 85 patients who were admitted to general wards before moving to the ICU

**Table S3** Proportion of RRT in AKI patients (N=508)

| **Site** | **AKI patients (n)** | **APACHE II** | **Number of RRT patients** | **Incidence of RRT (%)** | **95% CI (%)** |
| --- | --- | --- | --- | --- | --- |
| Mahosot hospital | 72 | 18.2 ± 7.6 | 0 | 0.0 | - |
| Mitraphap hospital | 175 | 19.2 ± 10.3 | 2 | 1.1 | 0.3 -4.5 |
| Savannakhet hospital | 111 | 9.9 ± 5.9 | 1 | 0.9 | 0.1 -6.2 |
| Champasak hospital | 60 | 16.4 ± 5.9 | 4 | 6.7 | 2.5 -16.6 |
| Luang Prabang hospital | 90 | 17.3 ± 5.4 | 19 | 21.1 | 13.8 -30.8 |
| All | 508 | 16.6 ± 8.6 | 26 | 5.1 | 3.5 -7.4 |

**Table S4** Patients characteristics stratified by RRT in severe AKI (N=299)

| - **Characteristics** | **RRT** | | **P value** |
| --- | --- | --- | --- |
|  | **No (N=275)** | **Yes (N=24)** |  |
| Age (years) | 59.3 (17.1) | 50.7 (19.0) | 0.02 |
| Female | 118 (42.9%) | 14 (58.3%) |  |
| Reimbursement |  |  | 0.10^1^ |
| - Government officer | 14 (5.1%) | 3 (12.5%) |  |
| - Out-of-pockets | 234 (85.1%) | 17 (70.8%) |  |
| - Social security, private insurances | 27 (9.8%) | 4 (16.7%) |  |
| BMI |  |  | 0.41^1^ |
| - Underweight | 19 (6.9%) | 2 (8.3%) |  |
| - Normal | 202 (73.5%) | 15 (62.5%) |  |
| - Overweight / obese | 54 (19.6%) | 7 (29.2%) |  |
| Primary diagnosis |  |  |  |
| - Cardiovascular diseases | 25 (9.1%) | 3 (12.5) | 0.48^1^ |
| - Renal diseases | 32 (11.6%) | 9 (37.5) | <0.001 |
| - Infectious diseases | 45 (16.4%) | 4 (16.7) | 1.00^1^ |
| - Gastrointestinal diseases | 28 (10.2%) | 2 (8.3) | 1.00^1^ |
| - Hematologic disease | 3 (1.1%) | 0 | 1.00^1^ |
| - Respiratory diseases | 44 (16%) | 5 (20.8) | 0.57^1^ |
| - Neurologic diseases | 24 (8.7%) | 0 | 0.24^1^ |
| - Endocrine diseases | 26 (9.5%) | 0 | 0.25^1^ |
| - Rheumatologic diseases | 1 (0.4%) | 0 | 1.00^1^ |
| - Oncologic diseases | 3 (1.1%) | 0 | 1.00^1^ |
| - Surgical related diseases | 44 (16%) | 1 (4.2) | 0.15^1^ |
| Comorbidity |  |  |  |
| - Hypertension | 102 (37.1%) | 11 (45.8) | 0.40 |
| - DM | 101 (36.7%) | 7 (29.2) | 0.46 |
| - CKD | 78 (28.4%) | 9 (37.5) | 0.35 |
| - CAD | 9 (3.3%) | 0 (0) | 1.00^1^ |
| - CVD | 9 (3.3%) | 0 (0) | 1.00^1^ |
| - Malignancy | 5 (1.8%) | 0 (0) | 1.00^1^ |
| AKI Etiology |  |  |  |
| - Sepsis | 58 (21.1%) | 3 (12.5%) | 0.43^1^ |
| - Renal hypoperfusion | 118 (42.9%) | 9 (37.5%) | 0.61 |
| - Toxin and poisoning | 0 | 1 (4.2%) | 0.08^1^ |
| - Trauma and surgery | 38 (13.8%) | 1 (4.2%) | 0.341 |
| - Genitourinary | 3 (1.1%) | 1 (4.2%) | 0.29^1^ |
| - Tropical infection | 2 (0.7%) | 0 | 1.00^1^ |
| - Obstetric complications | 3 (1.1%) | 2 (8.3%) | 0.05^1^ |
| - Others | 53 (19.3%) | 7 (29.2%) | 0.25 |
| AKI type^2^ | (N=230) | (N=15) | 1.00^1^ |
| - CA-AKI | 174 (75.7%) | 12 (80.0%) |  |
| - HA-AKI | 56 (24.4%) | 3 (20.0%) |  |
| APACHE-II score | 19.7 (8.3) | 19.5 (6.2) | 0.90 |
| Non-renal SOFA score | 6.0 (3.6) | 4.4 (2.5) | 0.03 |
| Vasopressors | 70 (25.5%) | 6 (25.0%) | 0.96 |
| Mechanical ventilation | 68 (24.7%) | 0 (0) | 0.002^1^ |
| Anemia | 154 (56%) | 21 (87.5%) | 0.003 |
| Urine output | 400 (200,1000) | 350 (125,800) | 0.34 |
| Fluid accumulation | 0.8 (0.15,1.53) | 1.22 (0.66,3.16) | 0.01 |
| Diuretics | 58 (21.1%) | 17 (70.8%) | <0.001 |

Data are mean (SD), n (%), or median (Q1,Q3), unless stated otherwise.

^1^ P value from Fisher’s exact test instead of chi-square test

All these parameters came from the first day of ICU admission

BMI: body mass index, DM: diabetes mellitus, CKD: chronic kidney disease, CAD: coronary artery disease, CVD: cerebrovascular disease, APACHE II: acute physiologic and chronic health evaluation II, SOFA: sequential organ failure assessment, CA-AKI: community-acquired AKI, HA-AKI: hospital-acquired AKI

^2^ Excluded 54 patients who were admitted to general wards before moving to the ICU

**Table S5**. Univariate and multivariate analysis of potential risk factors for renal replacement therapy (RRT) in patients with stage 3 (n= 299) using mixed logistic regression clustering by sites

| **Characteristics** | **Univariate** | **P value** | **Multivariate** | **P value** |
| --- | --- | --- | --- | --- |
|  | Unadjusted OR (95% CI) |  | Adjusted OR (95% CI) |  |
| Age, 10-year increment^1^ | 0.74 (0.55,0.98) | 0.038 | 0.72 (0.50, 1.03) | 0.07 |
| Primary diagnosis |  |  |  |  |
| - Renal diseases | 3.90  (1.26, 12.12) | 0.018 | 2.78  (0.71, 10.94) | 0.14 |
| AKI Etiology |  |  |  |  |
| - Obstetric complications | 10.31 (0.97,110.11) | 0.053 | 4.41  (0.22, 87.10) | 0.33 |
| APACHE-II score | 0.99 (0.92,1.08) | 0.89 |  |  |
| Non-renal SOFA score | 0.85 (0.67,1.07) | 0.17 | 0.84 (0.61,1.16) | 0.29 |
| Anemia | 4.42 (1.15,16.97) | 0.03 | 20.67 (2.07,206.15) | 0.01 |
| Fluid accumulation | 1.58 (1.15,2.17) | 0.005 | 2.10  (1.32, 3.34) | 0.002 |
| Diuretics | 3.91 (1.26,12.19) | 0.019 | 3.55  (0.73, 17.16) | 0.12 |

APACHE II: acute physiologic and chronic health evaluation II, SOFA: sequential organ failure assessment

**Table S6** Patients characteristics characterized by hospital discharge status in all AKI patients (n = 508)

| **Characteristics** | **Survive**  **(n = 207)** | **Die**  **(n = 226)** | **AMA**  **(n = 75)** | **P-value** |
| --- | --- | --- | --- | --- |
| Age (years) | 51 (33,66) | 59 (43.5,70)* | 59 (42,69)** | <0.001 |
| Male | 113 (54.6%) | 140 (61.9%) | 45 (60%) | 0.29 |
| Reimbursement |  |  |  | 0.05 |
| - Government officer | 16 (7.7%) | 10 (4.4%) | 5 (6.7%) |  |
| - Out-of-pockets | 158 (76.3%) | 198 (87.6%) | 61 (81.3%) |  |
| - SS, private insurances | 33 (15.9%) | 18 (8.0%) | 9 (12%) |  |
| BMI |  |  |  | 0.31 |
| - Underweight | 17 (8.2%) | 15 (6.6%) | 4 (5.3%) |  |
| - Normal | 136 (65.7%) | 169 (74.8%) | 53 (70.7%) |  |
| - Overweight/obese | 54 (26.1%) | 42 (18.6%) | 18 (24%) |  |
| Primary diagnosis |  |  |  |  |
| - Cardiovascular diseases | 23 (11.1%) | 24 (10.6%) | 1 (1.3%) | 0.07^1^ |
| - Renal diseases | 23 (11.1%) | 19 (8.4%) | 7 (9.3%) | 0.88 |
| - Infectious diseases | 22 (10.6%) | 32 (14.2%) | 23 (30.7%) | 0.008 |
| - Gastrointestinal diseases | 34 (16.4%) | 19 (8.4%) | 5 (6.7%) | <0.001 |
| - Hematologic disease | 5 (2.4%) | 3 (1.3%) | 1 (1.3%) | 0.26^1^ |
| - Respiratory diseases | 41 (19.8%) | 26 (11.5%) | 14 (18.7%) | 0.02 |
| - Neurologic diseases | 14 (6.8%) | 31 (13.7%) | 6 (8%) | <0.001 |
| - Endocrine diseases | 12 (5.8%) | 26 (11.5%) | 4 (5.3%) | 0.11 |
| - Rheumatologic diseases | 0 | 1 (0.4%) | 0 | 0.46^1^ |
| - Oncologic diseases | 2 (1.0%) | 4 (1.8%) | 3 (4%) | 0.16^1^ |
| - Surgical related diseases | 31 (15.0%) | 41 (18.1%) | 11 (14.7%) | 0.10 |
| Comorbidity |  |  |  |  |
| HT | 60 (29.0%) | 100 (44.3%) | 19 (25.3%) | 0.001 |
| DM | 46 (22.2%) | 90 (39.8%) | 24 (32%) | <0.001 |
| CKD | 57 (27.5%) | 47 (20.8%) | 17 (22.7%) | 0.25 |
| Cerebrovascular | 10 (4.8%) | 5 (2.2%) | 3 (4.0%) | 0.33^1^ |
| Malignancy | 9 (4.4%) | 9 (1.3%) | 3 (4%) | 0.15 |
| CAD | 8 (3.9%) | 4 (1.8%) | 2 (2.7%) | 0.41^1^ |
| AKI staging |  |  |  | <0.001 |
| 1 | 33 (15.9%) | 11 (4.9%) | 15 (20%) |  |
| 2 | 63 (30.4%) | 64 (28.3%) | 23 (30.7%) |  |
| 3 | 111 (53.6%) | 151 (66.8%) | 37 (49.3%) |  |
| AKI etiology |  |  |  |  |
| - Sepsis | 38 (18.4%) | 46 (20.4%) | 25 (33.3%) | 0.02 |
| - Renal hypoperfusion | 113 (54.6%) | 99 (43.8%) | 29 (38.7%) | 0.02 |
| - Toxin and poisoning | 1 (0.5%) | 0 | 0 | 0.56^1^ |
| - Trauma and surgery | 18 (8.7%) | 34 (15.0%) | 8 (10.7%) | 0.12 |
| - Systemic disease | 1 (0.5%) | 0 | 0 | 0.56^1^ |
| - Genitourinary | 5 (2.4%) | 1 (0.4%) | 2 (2.7%) | 0.12^1^ |
| - Tropical infection | 2 (1.0%) | 1 (0.4%) | 3 (4.0%) | 0.07^1^ |
| - Obstetric complications | 5 (2.4%) | 5 (2.2%) | 8 (10.7%) | 0.51^1^ |
| - Others | 24 (11.6%) | 40 (17.7%) | 0 | 0.14^1^ |
| AKI type^2^ | (N=88) | (N=126) | (N=31) | 0.018 |
| - CA-AKI | 61 (69.3%) | 105 (83.3%) | 20 (64.5%) |  |
| - HA-AKI | 27 (30.7%) | 21 (16.7%) | 11 (35.5%) |  |
| APACHE-II score | 13.5 (6.7) | 21.5 (9.3)* | 16.2(7.3)**,*** | <0.001 |
| Non-renal SOFA | 3.8 (2.2) | 8.1 (3.2)* | 5.4 (2.7)**,*** | <0.001 |
| Anemia | 109 (52.7%) | 124 (54.9%) | 40 (53.3%) | 0.90 |
| Vasopressors | 35 (17.0%) | 61 (27.0%) | 21 (28.0%) | 0.02 |
| Mechanical ventilation | 14 (6.8%) | 73 (32.3%) | 11 (14.7%) | <0.001 |
| RRT | 18 (8.7%) | 8 (3.5%) | 0 | 0.005 |
| Urine output (ml) | 1000 (700,1500) | 600 (200,1400)* | 1000 (700,1500)*** | <0.001 |
| Percent of fluid accumulation | 0.6  (-0.3,1.3) | 0.7 (0.2,1.2) | 0.7  (-0.4,1.4) | 0.07 |
| Diuretic | 72 (34.8%) | 20 (8.9%) | 18 (24%) | <0.001 |
| Hospital length of stay (days) | 7 (5,11) | 2 (1,5)* | 3 (2,6)** | <0.001 |

Data are mean (SD), n (%), or median (Q1,Q3), unless stated otherwise.

*p<0.05, non-survivors vs. survivors

**p<0.05, AMA vs. survivors

***p<0.05, AMA vs. non-survivors

^1^ P value from Fisher’s exact test instead of chi-square test

AMA: against medical advice, BMI: body mass index, DM: diabetes mellitus, CKD: chronic kidney disease, CAD: coronary artery disease, CVD: cerebrovascular disease, APACHE II: acute physiologic and chronic health evaluation II, SOFA: sequential organ failure assessment, CA-AKI: community-acquired AKI, HA-AKI: hospital-acquired AKI, RRT: renal replacement therapy

^2^ Excluded 85 patients who were admitted to general wards before moving to the ICU

**Table S7** Univariate and multivariate analysis of potential risk factors for death or HAMA in AKI patients (n = 508) using mixed logistic regression clustering by sites

| **Characteristics** | **Univariate** | **P value** | **Multivariate** | **P value** |
| --- | --- | --- | --- | --- |
|  | Unadjusted OR (95% CI) |  | Adjusted OR (95% CI) |  |
| Reimbursement |  |  |  |  |
| - Government officer | Reference |  |  |  |
| - Out-of-pockets | 0.76 (0.30,1.91) | 0.56 |  |  |
| - Social security, private health insurances | 0.89 (0.40,1.95) | 0.77 |  |  |
| Primary diagnosis |  |  |  |  |
| - Cardiovascular diseases | 0.52 (0.25,1.09) | 0.08 | 0.50 (0.21,1.16) | 0.11 |
| - Renal diseases | 0.81 (0.40,1.65) | 0.56 |  |  |
| - Infectious diseases | 2.30 (1.27,4.16) | 0.006 | 1.59 (0.77,3.27) | 0.21 |
| - Gastrointestinal diseases | 0.40 (0.21,0.78) | 0.005 | 0.60 (0.28,1.29) | 0.20 |
| - Hematologic disease | 0.49 (0.11,2.16) | 0.35 |  |  |
| - Respiratory diseases | 0.84 (0.49,1.44) | 0.52 |  |  |
| - Neurologic diseases | 1.49 (0.71,3.14) | 0.30 |  |  |
| - Endocrine diseases | 1.72 (0.76,3.88) | 0.19 | 1.13 (0.44,2.94) | 0.80 |
| - Rheumatologic diseases | NA |  |  |  |
| - Oncologic diseases | 3.17 (0.58,17.34) | 0.18 |  |  |
| - Surgical related diseases | 1.11 (0.61,1.99) | 0.74 |  |  |
| Comorbidity |  |  |  |  |
| HT | 0.98 (0.63,1.55) | 0.95 |  |  |
| DM | 1.88 (1.19,2.97) | 0.007 | 2.06 (1.20,3.52) | 0.008 |
| AKI type^2^ |  |  |  |  |
| - CA-AKI | 1.19 (0.74,1.89) | 0.48 |  |  |
| - HA-AKI | Reference |  |  |  |
| AKI Etiology |  |  |  |  |
| - Renal hypoperfusion | 0.62 (0.41,0.94) | 0.024 | 0.71 (0.43,1.18) | 0.19 |
| - Trauma and surgery | 1.29 (0.64,2.58) | 0.48 |  |  |
| APACHE-II score | 1.07 (1.03,1.10) | <0.001 | 1.05 (1.01,1.09) | 0.006 |
| Non-renal SOFA | 1.42 (1.28,1.57) | <0.001 |  |  |
| Vasopressors | 0.51 (0.31,0.85) | 0.009 | 1.28 (0.71,2.31) | 0.41 |
| Mechanical ventilation | 2.76 (1.42,5.36) | 0.003 | 2.60 (1.20,5.64) | 0.02 |
| AKI staging |  |  |  |  |
| 1 | Reference |  |  |  |
| 2 | 1.14 (0.58,2.23) | 0.71 |  |  |
| 3 | 1.30 (0.69,2.46) | 0.42 |  |  |
| RRT | 0.42 (0.16,1.09) | 0.074 | 0.42 (0.15,1.22) | 0.11 |
| Urine output | 0.99 (0.99,1.00) | 0.19 | 0.99 (0.99,1.00) | 0.15 |
| Percent of fluid accumulation | 1.16 (1.01,1.33) | 0.034 | 1.13 (0.95,1.34) | 0.16 |
| Diuretics | 0.55 (0.33,0.90) | 0.018 | 0.56 (0.32,1.01) | 0.052 |

HT: hypertension, DM: diabetes mellitus, CA-AKI: community-acquired AKI, HA-AKI: hospital-acquired AKI, APACHE II: acute physiologic and chronic health evaluation II, SOFA: sequential organ failure assessment, RRT: renal replacement therapy

^2^ Excluded 85 patients who were admitted to general wards before moving to the ICU

**Figure S1** Distribution of patients by maximum AKI stage for each hospital type. Y-axis represents the percentage of patients. X-axis represents hospital type. The number on top of the bars is the raw count.

137

80

44

472

162

70

15

480

**Figure S2** Kaplan-Meier survival curves for each AKI stage on hospital mortality after excluding hospital discharge against medical advice (n = 1,261; Log rank p < 0.001)

**
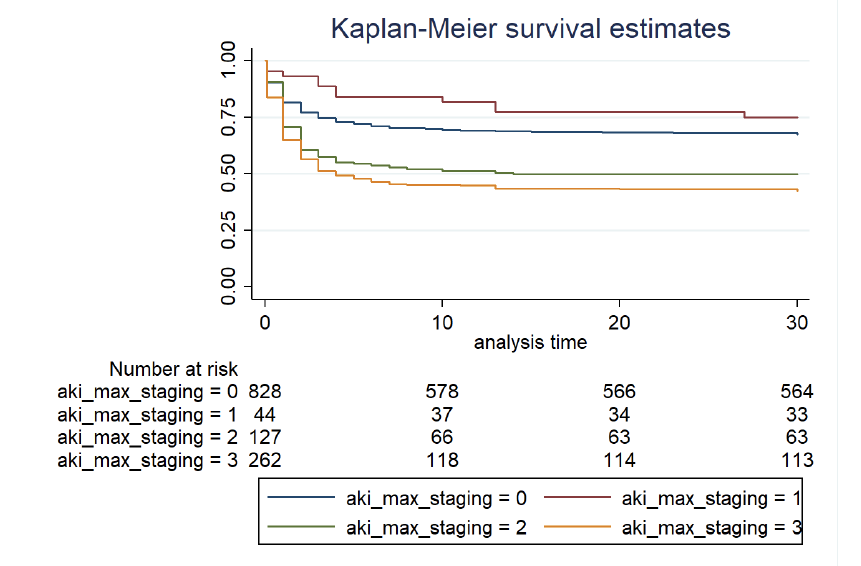
**

**Figure S3** Kaplan-Meier survival curves for each AKI stage on ICU mortality (n = 1,460; Log rank p < 0.001)

**
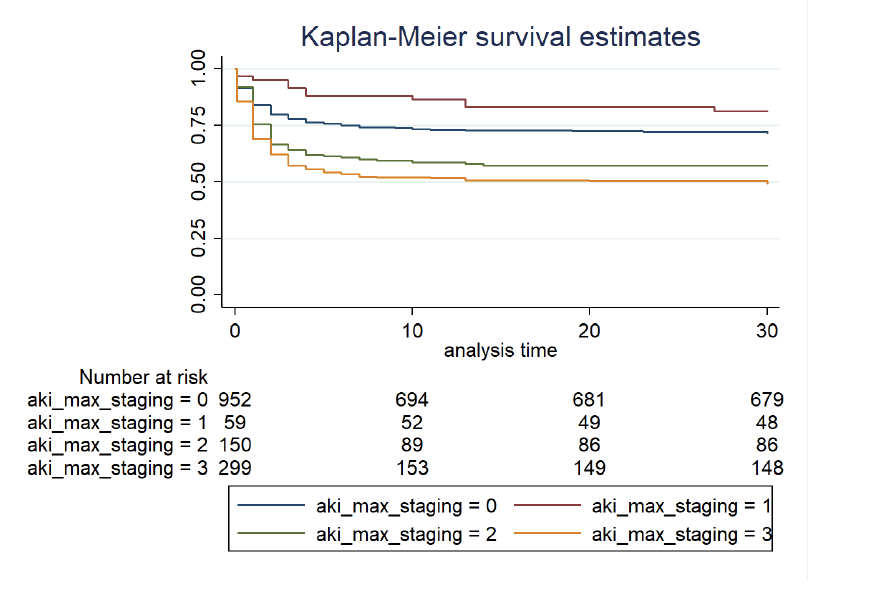
**

**Figure S4** Kaplan-Meier survival curves for each AKI stage on hospital mortality after excluding hospital discharge against medical advice (n = 1,261; Log rank p < 0.001)


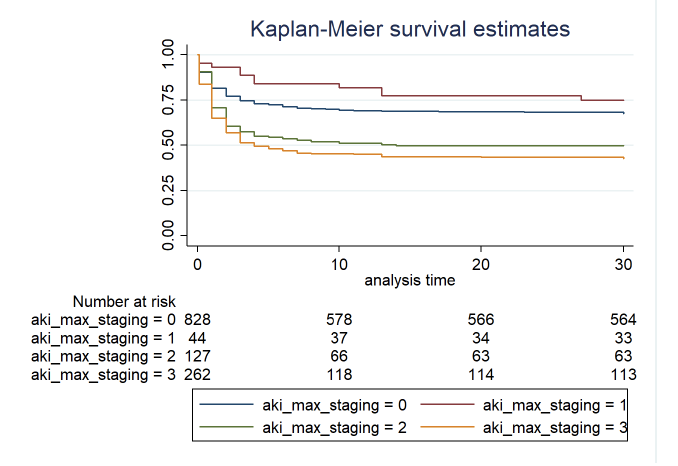

Supplement: Supplementary file 1 — Additional file 1: Table S1. Cumulative prevalence of AKI by sites (N=1,460). Table S2. Patients characteristics stratified by severity of AKI (N=508) using mixed logistic regression clustering by sites. Table S3. Proportion of RRT in AKI patients (N=508). Table S4 Patients characteristics stratified by RRT in severe AKI (N=299). Table S5. Univariate and multivariate analysis of potential risk factors for renal replacement therapy (RRT) in patients with stage 3 (n= 299) using mixed logistic regression clustering by sites. Table S6. Patients characteristics characterized by hospital discharge status in all AKI patients (n = 508). Table S7. Univariate and multivariate analysis of potential risk factors for death or HAMA in AKI patients (n = 508) using mixed logistic regression clustering by sites. Figure S1. Distribution of patients by maximum AKI stage for each hospital type. Y-axis represents the percentage of patients. X-axis represents hospital type. The number on top of the bars is the raw count. Figure S2. Kaplan-Meier survival curves for each AKI stage on hospital mortality after excluding hospital discharge against medical advice (n = 1,261; Log rank p < 0.001). Figure S3. Kaplan-Meier survival curves for each AKI stage on ICU mortality (n = 1,460; Log rank p < 0.001). Figure S4. Kaplan-Meier survival curves for each AKI stage on hospital mortality after excluding hospital discharge against medical advice (n = 1,261; Log rank p < 0.001). [file 12916_2020_1645_MOESM1_ESM.docx]
